# Impact of backscattered radiation from the bunker structure on EPID dosimetry

**DOI:** 10.1120/jacmp.v13i6.4024

**Published:** 2012-11-08

**Authors:** Pejman Rowshanfarzad, Mahsheed Sabet, Daryl J. O'Connor, Peter B. Greer

**Affiliations:** ^1^ School of Mathematical and Physical Sciences University of Newcastle Newcastle NSW 2308 Australia; ^2^ Department of Radiation Oncology Calvary Mater Newcastle Hospital Newcastle NSW 2310 Australia

**Keywords:** linac, EPID, dosimetry, bunker, backscatter

## Abstract

Amorphous silicon electronic portal imaging devices (EPIDs) have been investigated and used for dosimetry in radiotherapy for several years. The presence of a phosphor scintillator layer in the structure of these EPIDs has made them sensitive to low‐energy scattered and backscattered radiation. In this study, the backscattered radiation from the walls, ceiling, and floor of a linac bunker has been investigated as a possible source of inaccuracy in EPID dosimetry. EPID images acquired in integrated mode at discrete gantry angles and cine images taken during arcs were used with different field setups (18×18 and $10\times 10{\text{cm}}^{2}$ open square fields at 150 and 105 cm source‐to‐detector distances) to compare the EPID response at different gantry angles. A sliding gap and a dynamic head‐and‐neck IMRT field and a square field with a 15 cm thick cylindrical phantom in the beam were also investigated using integrated EPID images at several gantry angles. The contribution of linac output variations at different angles was evaluated using a 2D array of ion chambers. In addition, a portable brick wall was moved to different distances from the EPID to check the effect at a single angle. The results showed an agreement of within 0.1% between the arc mode and gantry‐static mode measurements, and the variation of EPID response during gantry rotation was about 1% in all measurement conditions.

PACS numbers: 87.56.bd; 87.56.J‐; 87.53.Bn; 87.56.Da

## I. INTRODUCTION

In modern radiotherapy techniques such as intensity‐modulated radiation therapy (IMRT) or intensity‐modulated arc therapy (IMAT), it is important to know the delivered dose with a high level of accuracy due to the steep dose gradients in the treatment plans;^1^, ^4^ therefore, it is necessary to quantify all possible sources of error in dosimetry measurements.

Electronic portal imaging devices (EPIDs) have been studied and used for dosimetry applications for many years.^5^, ^6^ EPIDs already exist in the structure of modern linacs and are therefore easy to setup. They have a large area of pixels providing a high‐resolution two‐dimensional array of real‐time digital data.^(7)^ The response of amorphous silicon (aSi) EPIDs is reproducible over short and long periods of time,^8^, ^10^ and is linearly related to dose.^8^, ^11^, ^12^ They have been used for verification of IMRT treatment plans^13^, ^14^ and for *in vivo* dosimetry measurements.^15^, ^16^ However, like any other dosimetry system, EPIDs have their own drawbacks. For instance, the presence of a gadolinium oxysulphide scintillator layer in the structure of aSi EPIDs has made them sensitive to low‐energy scattered^(12)^ or backscattered radiation.^(17)^ The effect of nonuniform backscatter from the EPID support arm in Varian linacs has already been investigated in several studies.^18^, ^21^ This led to the idea that there may also be an effect on EPID dosimetry measurements caused by the backscattered radiation from the treatment room structural components.

In the present study, the possibility of inaccuracies in EPID dosimetry as a result of the backscattered radiation from the treatment bunker walls is investigated and its level of importance is relatively evaluated. The presence of such an effect could lead to errors, not only in static measurement conditions, but also in dosimetry during arc deliveries, since the distance between the EPID detector and the surrounding walls continuously changes during arcs; therefore, it has been tested for both modes.

## II. MATERIALS AND METHODS

All irradiations were performed using 6 MV photon beams of a Varian Trilogy linear accelerator (Varian Medical Systems, Palo Alto, CA). EPID images were acquired in DICOM format using a Varian Portal Vision aS1000 EPID attached to the linac by an E‐type supporting arm. The EPID had an active area of $40\times 30{\;\text{cm}}^{2}$ containing 1024×768 pixels.

The bunker walls were constructed of ∼2 m thick conventional concrete $\left(∼2.4\;\text{gr}.{\text{cm}}^{‐3}\right)$ to provide adequate radiation shielding for 6 and 18 MV radiotherapy beams. The distance between the linac isocenter and the left wall, right wall, floor, and ceiling of the bunker were 370, 385, 130, and 145 cm, respectively.

The couch bearing system (for rotation) is installed in a cylindrical cavity (130 cm diameter and 30 cm depth) beneath a circular timber‐top. The structure of the floor is not homogeneous in this part, due to the presence of the steel bearing system of the couch and thick steel sub‐frames. The distance from the roof specified above is measured from the dropped ceiling of the bunker, which is made of mineral fiber (a low‐density material) mounted on a gridwork of metallic frames. The plenum space between the dropped ceiling and the structural concrete ceiling (~ 30 cm) provides room to conceal piping, wiring, and ductwork.

In order to investigate the effect of backscattered radiation from the bunker construction components during arc deliveries, EPID images were acquired using 1200 MU irradiations at a nominal rate of 600 MU/min in continuous (cine) image acquisition mode at a rate of 7.5 frames per second (6 frames per image). The EPID was positioned at 105 and 150 cm source‐to‐detector distances (SDD) during 360° gantry rotations, which yielded one image per ~ 2.5° rotation. Images were taken for 10×10 and $18\times 18{\;\text{cm}}^{2}$ jaw‐defined field sizes.

For static mode investigations, integrated EPID images were acquired at SDD=105 and 150 cm for eight gantry angles in 45° increments using 100 MU at a rate of 300 MU/min. The effect of backscatter was tested for open 10×10 and $18\times 18{\;\text{cm}}^{2}$ jaw‐defined fields, as well as a 2.5 cm wide sliding gap and a head‐and‐neck IMRT field to provide data for more realistic clinical treatment conditions.

The linac output variation with gantry angle was also tested, since it might be the reason for some of the differences observed in the EPID signal during gantry rotation. This was performed using a ${\text{MatriXX}}^{\text{Evolution}}$ two‐dimensional array of ionization chambers (IBA Dosimetry, Schwarzenbruck, Germany) fixed to the gantry head at 100 cm distance from the source using the head mount supplied by the manufacturer. The head mount was firmly attached to the gantry head to minimize the possibility of small movements during rotation and to hold the detector array perpendicular to the beam at all gantry angles. The detector array consists of 1020 vented $0.08\;{\text{cm}}^{3}$ ionization chambers arranged in a 32×32 grid in a $24.4\times 24.4\;{\text{cm}}^{2}$ area. Measurements with the MatriXX detector were made using 100 MU irradiations at a rate of 300 MU/min for $18\times 18{\;\text{cm}}^{2}$ jaw‐defined fields at eight gantry angles in 45° increments. The results were converted into DICOM format for processing and the central $9\times 9{\;\text{cm}}^{2}$ of each image was used as the region of interest.

More investigation on the effect of wall backscatter was carried out by independently measuring the effect. Eight brick blocks ($20\times 60\times 7.5\;{\text{cm}}^{3}$ dimensions, $\text{density}∼1\;\text{gr}.{\text{cm}}^{‐3}$) were walled up on a trolley, providing a $80\times 60\times 15\;{\text{cm}}^{3}$ brick layer which was easily moved to different distances (50, 75, 100, 150, 200, and 300 cm) from the back of the EPID. The EPID was positioned at SDD=100 cm with the gantry set at 270°, and integrated images were acquired with 100 MU irradiations at 300 MU/min.

Although the MatriXX detector system has a backscatter layer equivalent to 3.5 cm of water in its structure^(22)^ and it is therefore unlikely to be affected by backscattered photons, a similar experiment was performed with the wall moved behind the MatriXX.

In addition, a homogenous cylindrical polymethylmethacrylate (PMMA) phantom with a diameter of 15 cm was set up isocentrically in the beam along the gantry rotation axis. It was positioned off the end of the table to avoid the table artifacts. Changes in EPID response was investigated for transit dosimetry conditions using $15\times 15{\;\text{cm}}^{2}$ jaw‐defined fields.

In this study, three measurement series were acquired for each setup. Evaluation of the imager response was based on the pixel values from the central 50% of the fields in order to provide adequately large areas to capture the backscatter signal and to limit the region of interest to within the field, and thus eliminate the effects of any possible sagging of the EPID^(23)^ (or MatriXX) and collimator jaws^(24)^ during rotation. The field edges were determined by developing a code which used the image pixel values, picking the first and the last points with gray scale levels larger than 50% of the maximum signal in the cross‐plane and in‐plane directions. The region of interest was then limited to the central 50% of the field area.

Data analysis was performed using MATLAB programming language and software (The Mathworks Inc., Natick, MA).

## III. RESULTS

### A. Open square fields

The range of variations in EPID response (difference between the maximum values ± 1 SD for each data series) for open $18\times 18{\;\text{cm}}^{2}$ fields at SDD=105 cm and 150 cm and $10\times 10{\;\text{cm}}^{2}$ fields at SDD=150 cm in both arc and static modes are given in Table 1.

**Table 1:** Range of relative EPID response variations (± 1 SD) using open square fields in different measurement setups at various gantry angles during cine EPID imaging in a 360° arc and integrated EPID images at distinct gantry angles.

	$SDD=150\;cm,18\times 18\;cm^{2}\;Field$	$SDD=105\;cm,18\times 18\;cm^{2}\;Field$	$SDD=150\;cm,10\times 10\;cm^{2}\;Field$
Cine Mode, Arc Delivery	0.71%±0.02%	0.47%±0.03%	0.52%±0.06%
Integrated Mode, Gantry Static	0.77%±0.04%	0.54%±0.04%	0.52%±0.05%

**Figure 1:**
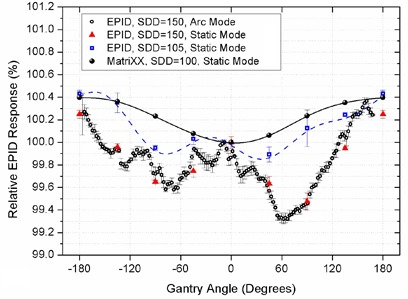
Relative EPID responses from $18\times 18{\;\text{cm}}^{2}$ fields using the integrated images acquired at discrete gantry angles in 45° intervals at SDD=150 cm and 105 cm, the cine images acquired during 360° arcs at SDD=150 cm, compared to the relative variations of linac output (measured by the MatriXX) at discrete gantry angles.

Variations in EPID signal at SDD=150 and 105 cm using $18\times 18{\;\text{cm}}^{2}$ fields are shown in Fig. 1 using integrated images acquired at discrete gantry angles in 45° intervals. In addition, the signal from cine EPID images acquired in arc mode at SDD=150 cm using $18\times 18{\;\text{cm}}^{2}$ fields are plotted for comparison.

One possible source of changes in EPID response at different gantry angles could be the small variation in the linac output, which could be caused by small movements of the gantry head components which cause changes in the beam path.^(25)^ Results of the MatriXX two‐dimensional dosimeter measurements at eight gantry angles in 45° increments are also given in Fig. 1.

The average results of three measurement series for each condition are used for Fig. 1, and the data points in each series are normalized to the EPID response at zero gantry angle. Curves are fitted through each series of data points for easier visual comparison.

Figure 1 shows that the variations in EPID response with gantry angle follow different patterns from the MatriXX measurements (which represent linac output variations). The range of output variation with gantry angle was ~ 0.4%.

### B. Portable brick wall

**Figure 2:**
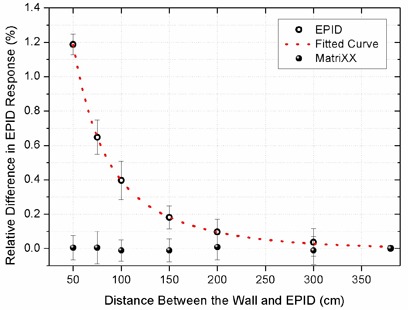
Relative differences in EPID and MatriXX signal as a result of placing a brick wall at 50, 75, 100, 150, 200, and 300 cm distances from the back side of the detector cassette, compared to “no brick wall” condition (used as reference).

Changes in the EPID and MatriXX response due to the backscatter from a brick wall moved to different distances from the detector are shown in Fig. 2. The percentage relative difference of each measurement with the reference condition of “no brick wall” (just the bunker wall) is plotted as a function of the distance between the wall and the detectors.

Figure 2 clearly shows the effect of low‐energy backscattered radiation from the brick wall on the EPID signal. The decrease in signal follows a double exponential curve.

The MatriXX response remained unaffected by the presence of the wall, as expected. Therefore, any variation in MatriXX measurements at different gantry angles could be attributed to variations in the linac output.

### C. Sliding gap and IMRT fields

**Figure 3:**
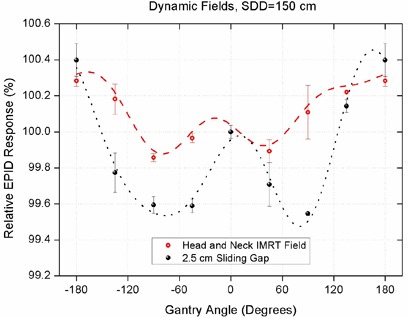
Variation of EPID response using images acquired at discrete gantry angles in 45° intervals at SDD=150 cm for a 2.5 cm wide sliding gap and a head‐and‐neck dynamic IMRT field.

Although measurements with square fields could be used to reveal the backscatter effect, it was necessary to investigate it in more realistic clinical conditions where the shape and size of the aperture change during the beam delivery. Changes in EPID response as a result of gantry rotation were tested for a simple 2.5 cm wide sliding gap moving across a $18\times 18{\;\text{cm}}^{2}$ field and also for a clinical head‐and‐neck dynamic IMRT field. Measurements were made at static gantry angles in 45° intervals and all data points were normalized to the EPID response at zero gantry angle. Results are shown in Fig. 3.

Variations in EPID response for the sliding gap and head‐and‐neck IMRT field at different gantry angles had a range of 0.85% ±0.05% and 0.43% ±0.02%, respectively.

### D. Transit measurements

**Figure 4:**
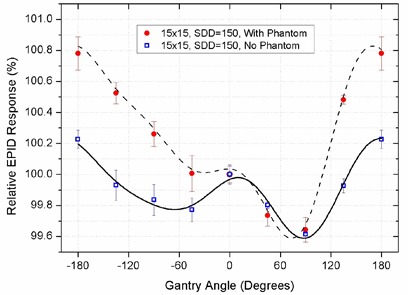
Variation of EPID response in transit conditions; the relative EPID responses from the images taken at discrete gantry angles in 45° intervals in presence of a cylindrical phantom in the beam are compared with “no phantom” conditions.

The presence of a phantom could affect the beam characteristics and modify the quality of backscattered radiation from the bunker. This effect was tested by placing a homogeneous cylindrical phantom isocentrically in the beam. The EPID responses in a $15\times 15{\;\text{cm}}^{2}$ field at discrete gantry angles in 45° intervals are compared with nontransit conditions (no phantom in the beam) in Fig. 4.

The range of the relative EPID response variations (± 1 SD) was 1.14% ±0.11% when measurements were performed in presence of the phantom, and 0.61% ±0.03% when there was no phantom in the beam.

## IV. DISCUSSION

Considering the importance of highly accurate dosimetry measurements for advanced radiotherapy treatments and due to the increasing application of EPID dosimetry techniques, it is important to understand the EPID dosimetry system characteristics and identify all possible sources of error in these measurements and quantify their effects. It has already been shown that EPIDs are sensitive to low‐energy radiation, and therefore the backscattered radiation from the treatment room structural components may have an effect on EPID dosimetry measurement results. The present study was conducted to examine the existence of such an outcome and the extent of its influence.

Results of this study showed that the EPID response varied at different gantry angles and distances from the walls. According to Fig. 1, the results observed for variation of the EPID response could not simply be attributed to variations in the linac output at different gantry angles. In fact, the output variations moderated the backscatter effect to some extent. However, since the final effect for EPID dosimetry in patients is a combination of the output variations and the backscatter from the walls, separate evaluation of these effects was not required.

Although sufficient data were provided to prove the existence of the effect of low‐energy backscattered radiation from the bunker structure on EPID signal, results of the measurements with the portable brick wall provided clear evidence for this effect. Due to the lower density and thickness of the blocks (compared to the bunker walls), the method was unable to exactly produce the same amount of increase in the EPID signal, but was capable of revealing the presence of the effect.

The photon energy spectrum of a 6 MV beam peaks at about 1 MV and has lower intensity at higher energies.^(26)^ At this energy range, Compton scattering is the predominant interaction of the beam with both conventional concrete and steel, which were used as the main building material and for system installations, and have effective atomic numbers of 12.5 and 26.0, respectively. Since the EPID detector has a $40\times 30{\;\text{cm}}^{2}$ active array, the portion of backscattered radiation from the bunker building structures which are at angles between ~ 175° to 180° relative to the incident rays, can mainly be detected. According to the well‐known formula for Compton scattered photon energies, the energy of the majority of backscattered photons from the walls would be in the range of 200 to 250 keV. Amorphous silicon EPIDs are known to be more sensitive to this range of energy due to the presence of a high atomic number phosphor scintillator layer in their structure.^27^, ^28^ This leads to changes in EPID response at different gantry angles and different distances from the walls, as shown in Fig. 1.

According to Table 1, increasing the size of the radiation field from $10\times 10{\;\text{cm}}^{2}$ to $18\times 18{\;\text{cm}}^{2}$ leads to an increase of about 0.2% in the backscatter effect from the bunker construction components. This phenomenon is attributed mainly to the larger interaction area of the wall, and partly to the decrease in the mean energy of the beam for larger fields due to the contribution of more low‐energy photons from the head components.^(29)^ It must be noted that the range of EPID response variations was less than 1% even for the large $18\times 18{\;\text{cm}}^{2}$ fields at 150 cm SDD. However, the effect of backscatter from the bunker walls was not limited to open square fields and was also present in dynamic dose deliveries, as shown in Fig. 3, with a range of less than 1% variation in EPID response.

Another point to consider is the possibility of changes in detector position (EPID sag) in the beam direction during arc deliveries. This has already been discussed in detail in a previous study^(23)^ and, based on those results, the average EPID sag effect on EPID response would not be larger than 0.1%.

The backscatter effect was also detected in transit dosimetry conditions. The presence of phantom leads to changes in beam characteristics due to the removal of low‐energy head‐scattered photons. However, it also results in the production of phantom‐scattered radiation which has lower energies compared to “no phantom” conditions. These photons have a higher probability of Compton scattering with the walls since the probability of Compton effect is inversely related to the beam energy. As a consequence, the presence of a phantom leads to the production of a larger amount of backscattered radiation from the wall with lower energies. As the EPID is more sensitive to low‐energy radiation, the relative EPID response in presence of the phantom has a larger range of variations (Fig. 4).

Variations to the reported values in this study are expected, depending on the size and construction of the bunker, but the order of magnitude is not expected to change much as ceiling and floor are often at comparable distances.

Addition of a lead sheet to the back of a Varian EPIDs has already been suggested in previous studies to reduce the nonuniform effect of arm‐backscatter on EPID response.^18^, ^19^, ^30^ This method would also be able to effectively remove the low‐energy wall‐backscatter–induced dosimetric inaccuracies. Other manufacturers that do not have the problem of arm backscatter may use a lower density metal sheet to impose lighter weight on their systems.

## V. CONCLUSIONS

The impact of the backscattered radiation from the walls, ceiling, and floor of the bunker was expected to be very small, but it was worthwhile to perform a systematic and quantitative study on the subject. This study showed that the effect can be ignored altogether for pretreatment verifications with the imager panel at the isocenter (SDD=100 or 105 cm), but the effect gradually increases with increasing SDD, and even more so when the larger SDD is combined with transit dosimetry. Fortunately, even in this ‘worst case scenario’, the effect still remains limited to 1% at its maximum.

## ACKNOWLEDGMENTS

This work was supported by the National Health and Medical Research Council Grant (Grant No. 569211). The authors wish to thank Dr. Patricia Ostwald and Mr. Dennis Pomare for their assistance in providing information about the bunker design and structure. The first author gratefully acknowledges the award of the UNIPRS scholarship from the University of Newcastle, Australia.
